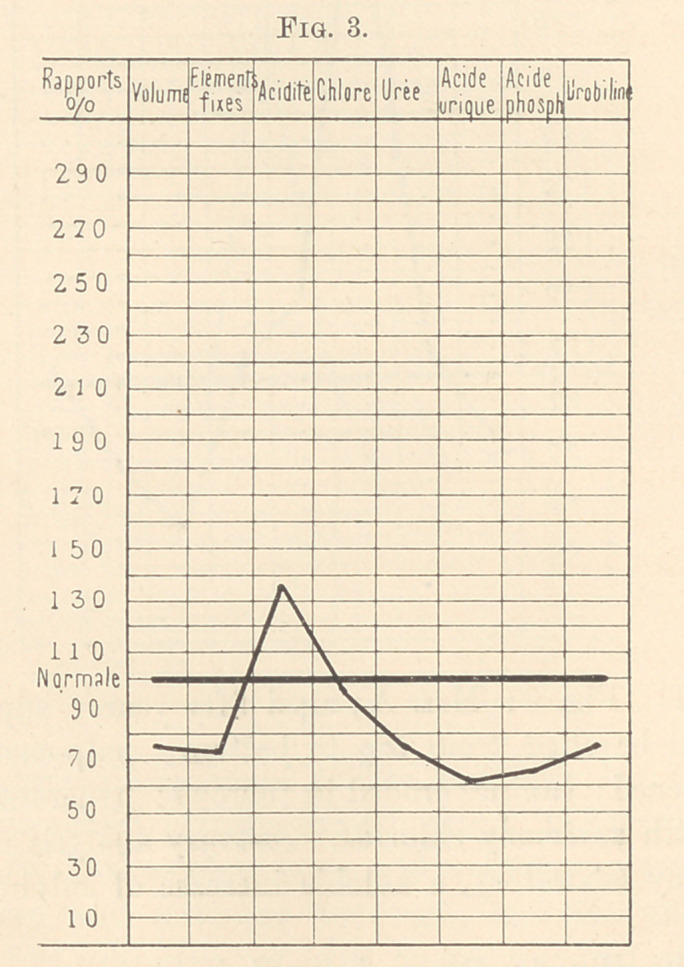# On the Rôle of Systemic Hyperacidity, and of Sulphocyanides in the Saliva, in Chemical Abrasion of the Teeth

**Published:** 1900-05

**Authors:** 


### Abstracts and Translations.

### ON THE RÔLE OF SYSTEMIC HYPERACIDITY, AND OF SULPHOCYANIDES IN THE SALIVA, IN CHEMICAL ABRASION OF THE TEETH.[1]

BY M. MICHAELS.[2]

(Continued from page 256.)

According to what has been already said concerning diatheses in general and their alterative effects upon the organism, it is evident that the cause of the lesion described must be sought in the saliva. But the salivary fluid is a complex, made up of the excretion of many glands,—parotid, submaxillary, sublingual, lingual,

---

[1] Paper read before the National Dental Congress of France, at the session at Nancy, August, 1898. Translated for The New York Institute of Stomatology by Drs. E. A. Bogue and C. O. Kimball, New York City.

[2] Honorary President of the National Dental Congress.

buccal, and labial. It is of the last, the labial glands, that we must say a few words, for the position of their excreting ducts seems to explain the peculiar localization of the erosion.

The labial mucous glands are situated between the muscular layer of the lip (the articularis oris) and the mucous membrane; they are very numerous, and describe a complete ring about the buccal orifice. More numerous upon the lateral portions of the lips than either at the commissures or in the middle, these glands lie in the thickness of the connective tissue under the mucous membrane, and are surrounded by fatty tissue. Each gland consists of several acini, and is provided with an excretory duct which widens at its lower extremity and opens upon the mucous surface in the vestibular cavity. Often the excretory duct receives other ducts from accessory glandules. To examine these glands the lip may be reversed and dried, and after a short interval a little drop of clear secretion gathers at each duct opening.

The mixed saliva produced by all the glands is a slightly alkaline fluid which contains a special ferment, ptyalin, in the proportion of seven parts per thousand.

Besides mucin, saliva contains chlorides of sodium and potassium in varying amounts, sulphates of sodium and potassium, earthy phosphates and carbonates, phosphate of iron, fatty matters, and derivatives of acid urates; these are constant elements. But in certain conditions we may find also urea, glucose, biliary pigment, lactic acid, and leucine, and these substances, except urea, are found only in pathological conditions.[1]

I have no doubt that the functional activity of the glands dialyzes all these chemical elements out of the blood plasma. Dissolved in water they are easily recognized, if not by direct analysis, then, after dehydration, by the microscope, or by the polariscope with transmitted light. Certain crystals are volatile, or change in form, or lose their color on drying; but it is possible to fix them and cover them in time with Canada balsam.

Certain crystalline organic substances when in solution will crystallize out in varied forms, branched, stellate, coral-like, or

[1] The reaction of the saliva may at times be freely acid, at other times amphoteric. In patients with hyperacidity, if turmeric paper be employed, the acidity may always be detected on carefully drying the paper. This double reaction occurs also in urine. (Traité d'analyse, etc., Gorup-Besarez.)

in amorphous and atypical shapes; and no treament is necessary to produce these crystals except evaporation of the fluid, and they are often beautiful and characteristic. This fact appears important and interesting. Profound micro-polariscopic study [1] of the chemical composition of the saliva would enable us to recognize initial steps in a developing disease, and urinary analysis would confirm such results.

It is well known that crystalline elements of the urate series are at times present in the saliva of hyperacid patients, in whom dental effects of their condition have been unrecognized, as well as the fact that excess of such chemicals is peculiar to certain pathological conditions. Derivatives of the acid urates are found in the secretions of hyperacid patients (gouty and rheumatic), the series of the sulphocyanides of sodium, ammonium, and potassium, and the series of acid and basic oxalates.

Among patients with diminished acidity the creatin group is found, and thereby special effects are produced on dental caries; for the carious process is then extremely rapid, and I have two cases, occurring in young subjects, where the teeth have been lost in spite of all the efforts of specialists.

My investigations of saliva compel me to recognize other causes than micro-organisms for dental caries. The chemical influence of the saliva is undeniable, as can be proved by any physiologist or chemist who will respect my observations. The objections of certain authors, who deny that saliva contains acid enough to produce appreciable effects, seem to me to rest either upon experimental errors or upon lack of reflection. Various medicines introduced into the body reappear in the saliva; iodides, for instance, occur as alkaline iodides, bromides act similarly, mercury also,—in short, one may justly conclude that active glands absorb, secrete, and excrete other things than their normal products. No matter how feeble the dissolved chemical element may be, if it have affinity for a base, there is a reaction. These reactions may be more manifest, or less; that is merely a question of the proportions present and the length of contact.

The crystalline characters of a chemical element vary according

---

[1] The apparatus for microscopy with polarized light consists of two Nichols prisms; one, the polarizer, is placed beneath the stage of the microscope, and the other, the analyzer, is mounted on a hood that fits over the eye-piece. (Nachet.)

to the proportion of acid and of base present; as in the case of potassium oxalate, which in complete crystallization is regular but may also appear, not completely crystallized, in branching forms.

Synthetic urates of urea and uric acid are well developed and easily found when the diathesis (hyperacid) is well marked; they exist also in saliva, and if not found it is owing to imperfect testing or observation, and they give rise to a series of changes in the hard tissues.

Salts of soda, chlorate of ammonium, sodium chloride lactates, tartrates, oxalates, sulphocyanides, and glucose in unstable combination in blood plasma and excretory fluids, are all detected by simple drying, and may be studied by the microscope.

It is from my study of the crystalline elements in the saliva that I have arrived at the opinion that this fluid should be considered in its action upon the teeth, for it is the excess of the synthetic compounds of uric acid in the saliva which is the morbid element in certain pathological states.

Acidity of the saliva is a local expression of a constitutional condition, and by precipitating mucin tends to form deposits of sordes on the teeth; this causes a peculiar form of caries of the neck of the tooth, so characteristic that I have no doubt every practitioner recognizes it.

The rheumatic taint explains still other appearances of the oral tissues. Certain affections of the gums, gouty deposits in the jaw-bones, alveolo-dental abscess, degeneration of the ligaments, painless loss of the teeth, are all results which accompany the gouty constitution.

Among the rheumatic the characteristic changes are different; salivary acidity is dependent on the sulphocyanides of sodium and ammonium, while the local manifestations are alveolar neuralgia, nervous constriction of the palate, diminished saliva, the teeth "set on edge," pain in the maxillary joint, penetrating caries, and chemical erosion of the teeth.

The special forms of oral changes due to glucose in the saliva are so distinct that we need only mention the softened and bleeding gums, the stale or fetid breath, and the denuded teeth. Diabetes is a hyperacid diathesis, and the manifold buccal affections of patients suffering from the disease are constantly and almost always the accompaniment of other general alterations. I ought to mention, however, that I have observed loss of the teeth as the initial stage of this disease. The fact is well known among medical men, and the analysis of the urine decides the diagnosis at once.

The changes observed in the teeth and the mucous membrane of the mouth are so clearly distinguished in different diatheses, that one must recognize a special salivary activity in each to explain the facts.

Fatty and uric acids, lactic and oxalic acids, acetone, and sulphocyanides are the acid matters which have a great affinity for the lime in the teeth. These acids occur in saliva in variable percentages, which explains the relatively long time needed to produce some of the effects.

The mistaken or unknown facts of vital chemistry, and the unrecognized relation of the saliva to each diathesis, result in the non-differentiation of these various acids and bases. Beaunis, in his treatise on Human Physiology, states that the presence of sulphocyanides in saliva is not constant, and that the conditions of their appearance are undetermined. He adds that they are thought to occur only in dental caries and in the mouths of smokers, although their formation has been clearly demonstrated in conditions other than these. According to him the mode of formation of sulphocyanides [1] in the body is unknown, but it may probably be explained by the presence of a molecule of cyanogen in the albuminous molecule. It is supposed, he declares, that sulphocyanides are only the result of disassimilation.

Gautrelet,[2] who is a biological chemist, states that the physiological excretion of sulphocyanic acid is considerable, and may be considered the chief regulator of the salivary glands, and hence of their digestive action upon starchy foods. The presence of sulphocyanides in the saliva may be detected by the addition of perchloride of iron.

But since derived uric acid combines with different bases,[3] as

---

[1] Potassium sulphocyanide may be formed synthetically as follows: Neutral adenin is changed by nitric acid to hypoxanthin, and potassium hydrate with this gives potassium sulphocyanide, $C_5H_5(NH)_5 + H_2NO_3 = C_5H_4(NH)_2O + 2(NH) + H_2O$, and $C_5H_5(NH)_5 + KOH = 5C(NH)K + 6H_2O$.

[2] See Gautrelet, "Urines, dépôts, sediments, etc."

[3] To detect faint traces of sulphocyanides in saliva or other organic liquids, which give no appreciable color with perchloride of iron, distil the ammonium, potassium, and sodium, the exact combination in any pathological state should be determined in order to distinguish it from others.

Among patients with acid excess these salts are constant but of variable quantity, and perchloride of iron does not distinguish the base combined; hence mistaken conclusions are possible. It is necessary, therefore, to use other tests,—Nessler's, for instance, —to detect ammonium, platinum bichloride for potassium, etc.

In the numerous urine analyses made for me by Gautrelet, among hyperacid patients especially, to determine their characteristic dental changes, we have had opportunity to analyze also the saliva of some cases of chemical erosion of the teeth; and Gautrelet states that the sulphocyanide is an ammonium, not a potassium salt, as the physiologists claim.

According to my observations and analyses, sodium and ammonium sulphocyanides are easily detected in the saliva of most hyperacid patients, and the microscope also discovers them; but there is never any chemical abrasion of the teeth.

I have determined that rheumatic patients have a characteristic form of caries, of a blackish tint, attacking the neck of the tooth. To clear up the question, M. Monfet, the chemist, has analyzed for me the saliva of a case where there was chemical erosion of several teeth; and it was discovered that the sulphocyanide was a double salt of ammonium and potassium, and it is this latter element which, in my opinion, is the active cause of the peculiar destruction mentioned. The analysis of the urine demonstrated, further, a condition of rheumatism and neurasthenia. It contained abundant calcium oxalate, incompletely crystallized, the total acidity expressed as phosphoric acid was increased two hundred and sixty-one per cent., and the urea was only seventy-nine per cent.

In three other cases presenting chemical erosion the urine gave a similar analysis; there were abundant leucomaines and creatin, the calcium oxalate is constant, the acidity expressed as phosphoric acid is increased from one hundred and thirty to two hundred and ninety-five per cent.; the lower figure, acidity one hundred and

---

saliva with phosphoric acid, try the first drops which come over with filter paper dipped in perchloride of iron solution, to which has been added hydrochloric acid, and then dried; each drop of the distilled saliva gives a red stain. (Beaunis, Phys. Hum., ii. p. 27.)

thirty per cent., in a case of rheumatic migraine, was due to the fact that all the normal elements were reduced.

The salivary analysis in the same case gave 0.072 gramme alkaline sulphocyanides to the litre, or about five hundred per cent., and the salivary acidity, in phosphoric acid, was two hundred per cent. above normal. The average urinary acidity among the rheumatic varies from two hundred to three hundred per cent.

The characteristic features of the rheumatic diathesis, then, in my understanding of it, are diminished urine (from sixty to seventy-five per cent. of normal daily amount), and diminution of all normal urinary constituents together, and appearance in the saliva of uric acid derivatives, sulphocyanides, and oxalic acid, varying from two to seven times the normal.

Chemical abrasions of the teeth are very clearly circumscribed, and are due to constant contact of secretions from the labial glands; but the question remains as to what chemical agent dissolves the dental enamel and causes such destruction. In view of the numerous products which I have discovered in the saliva, already mentioned above, I have endeavored to reproduce chemical erosion experimentally.

The hypothetic action of alkaline sulphocyanides (of potassium and ammonium) is as folllows: They dissolve the ossien [1] of the teeeth, expose their mineral elements, and unite with them to form sulphocyanide of calcium and soluble phosphates of potassium and ammonium.

The following has been my method of experiment: Twelve cubic centimetres of saliva, freshly drawn, are filtered and acidulated with one drop of hydrochloric acid. The fluid is then divided into two portions. Of these, one is concentrated by gentle heat after a few drops of an alcoholic solution of platinum chloride are added, and at the end of four or five days octahedral crystals are developed, yellow in color, composed of platino-chlorate of ammonium and potassium. The other portion is evaporated to dryness in a platinum crucible and calcined at a dull red heat. The

[1] The organic principle which solidifies the prisms of enamel is keratine, a special chemical constituent of epithelial tissues, such as hair, nails, horn; and found also in non-epithelial membranes, such as gland capsules, the crystalline lens, sarcolemma, and neurilemma, and cell membranes of cartilage, bone, and connective tissue. The mode of formation is unknown. It is insoluble in alcohol and ether, swells up with acetic acid, and dissolves in caustic alkalies.

sialoin and ammoniacal salts are thus volatilized. A little distilled water, containing a few drops of alcoholic solution of platinum, is then added, and the mixture set aside. After four or five days, crystals slowly form, less abundant than in the other portion, entirely composed of potassium platino-chlorate, in straw-colored octahedral crystals; these are easily discovered by the microscope.

The patient whose saliva was thus investigated was my patient for several years, and in July, 1896, my attention was drawn to his case. On my request, Gautrelet, at Vichy, analyzed both his urine and saliva. The results were: Saliva: reaction neutral; contains ammonium sulphocyanide, 0.120 cubic centimetre per litre. Compare this with mixed saliva: 0.050 cubic centimetre per litre; and Jakowsky's figures: 0.060 cubic centimetre per litre. In other words, the saliva contained twice the normal amount of sulphocyanide, which in this case was only the ammonium salt, contrary to what I have found in normal saliva.

The urinary analysis by Gautrelet, of the same date, presents the special features of rheumatic arthritis; the urine was of high specific gravity, its fluorescence was increased, leucomaines abundant, and sulphocyanides in traces; abnormal element, calcium oxalate. The principal percentages were:

| | | Per cent. |
|---|---|---|
| Daily amount, 1200 cubic centimetres; normal for this case, 1776 cubic centimetres | | 73 |
| Acidity in phosphoric acid | 2.22 | 261 |
| Chlorine, as chlorides | 7.40 | 120 |
| Urea | 33.30 | 79 |

Another analysis made in June gave about the same result, but the acidity was less, being one hundred and fifty-eight per cent.; calcium oxalate, crystalline and amorphous, very abundant.

The following case seems to deserve special consideration: Mrs. A., fifty years of age; superior incisors eroded and bevelled from top to bottom; chemical action, so intense that the dentine is bare over the whole surface. The lateral incisors have a layer of secondary dentine covering in the pulp-cavity. The canines are eroded in ridges, the affected part being fan-shaped and sensitive to heat and touch. The mucous membrane of the upper lip presents confluent salivary glands whose secretion bathes the whole surface of the teeth.

This case, remarkable for the extent of the trouble (Fig. 3), is also of interest because of the severe pain it caused. The sensitiveness of the abraded surfaces was so acute that the patient was often obliged to keep to her bed. An anodyne treatment helped the condition until I decided to employ the potential cautery (chemical); the cure was immediate.

I have only the salivary analysis in this case. The reaction of the sulphocyanides was feeble, but, on the other hand, there was a strong ammoniacal reaction.

The hyperæsthesia varies much in different subjects of chemical erosion. It may be considerable in one case and absent in another, but when the abrasion results mechanically, from mastication, metallic supports for teeth, or too vigorous use of the toothbrush, there are periods of increased pain at times. The old method of actual cautery with the red-hot iron was painful and rather frightful, and chemical caustics like silver nitrate and gold chloride are not very powerful, and discolor the teeth. I have employed antimony chloride [1] with advantage, but as the caustic power of this salt is extreme, the greatest care is required in its use. Such therapeutic use of it, however, is efficacious and free from inconvenience with certain precautions, and it does not discolor the teeth. The pain of its application lasts but a moment and is easily borne, but where there are several abrasions present I treat only three or four at a sitting. I apply the caustic by raising the lip and protecting it with a roll of cotton, drying the surface of the tooth, and with the dull end of a quill toothpick rubbing on a drop of the antimony chloride. I avoid touching the gums, and over the spot I lay a small piece of some protective and retain it several seconds. Lastly, the mouth is rinsed with soda solution, four grains of the bicarbonate to the litre of water. While this caustic action may be severely painful, such effects last only a minute or two at most, and where a slight sensitiveness persists for a few days the treatment should be repeated. But usually once is enough,

---

[1] Antimony chloride is a poisonous caustic which disorganizes the tissues, and under the name of butter of antimony has been used by physicians to cauterize wounds made by the bites of rabid or poisonous animals. It destroys at the point of contact, but only superficially, but I think it has a deeper effect in its tendency to coagulate and mummify the albumin of the fibrillary neurin. The cure is radical and without return. Experimentally one can put a little white of egg in a test-tube, and add a drop of chloride of antimony; with a hand lens the coagulum is discovered at the point of contact, and all about it is a zone of its influence, where the albumin has been rendered non-putrescible.

Fig. 2.

Fig. 1.

Fig. 3.

Fig. 4.

Fig. 5.

Types of chemical abrasion of the teeth.

in the manner described, which is also to be recommended in any case where the dentine is denuded.

The prophylaxis of chemical abrasion is twofold, local and general. The destruction of the teeth may be arrested by puncturing the labial glands with the Paquelin thermo-cautery. These glands are very small and not deep, reaching in only one and a half millimetres below the mucous surface, and very slight cauterization will destroy them. Moreover, these hyperacid patients should be put on general alkaline treatment. And when the derived uric alkaloids and the sulphocyanides become overpowered by the total alkalinity, the destructive action ceases.

As my hypothesis of a chemical action to explain these cases of dental erosion may be received with doubt, I made the following experimental research to confirm it, and repeated it two or three times:

In a litre of water I dissolved one gramme of potassium sulphocyanide, which is greater by far than the normal percentage of the salt in the saliva; but as chemical erosion in the mouth may take five to ten years, one is justified in using a stronger solution for the test. A capillary tube, drawn out to a fine point and curved like the letter V, was passed through a cork and one end dipped into the sulphocyanide solution. The other end carried a wire which held a natural tooth against its tip, and this capillary siphon, once started, kept the tooth surface constantly wet. The movement of the fluid was, of course, very slow, but from time to time a drop would fall from the tooth into a receptacle below, and at the end of some days the surface of the enamel presented erosions entirely comparable with those of several in the mouths of patients.

In all my cases, the hyperacid had ammonium sulphocyanide in the saliva, but no abrasions; while those whose teeth were thus affected presented in their saliva the sulphocyanide of potassium.

Therefore, by reason of my observations, experiments, and analyses, I feel that my hypothesis, which I have advanced to explain chemical dental erosion, a problem for so long even to distinguished physiologists, has been demonstrated a verity.

NOTE.

Case I., Fig. 1: M. L., aged forty-eight years; lower teeth, as well as upper, present chemical erosion. The lower incisors, especially the left lateral incisor, are more involved. It is remarkable that the process has destroyed the dentine under the enamel of the teeth of two millimetres. One tooth alone of the upper arch shows a small abrasion in the centre of its surface. Gautrelet's analysis of the saliva in this case shows: Reaction neutral; sulphocyanide (the ammonium salt), 0.120 gramme to the litre; normal mixed saliva, 0.050 gramme to the litre; Jakowsky's analysis, 0.060 gramme to the litre. The saliva thus contains twice the normal percentage of sulphocyanide, and it is not the potassium but the ammonium salt.

Fig. 1.

The urine presents increased acidity and chlorides; other normal elements decreased, calcium oxalate, skatol, and peptones. The total acidity expressed as phosphoric acid is 4.80 grammes per litre, or two hundred and sixty-one per cent., and the chlorine (as chlorides) is 7.94 grammes per litre, or one hundred and twenty-eight per cent.

Case II., Fig. 2: M. B. M., aged forty-two years; three of the upper teeth are abraded; the right canine is almost destroyed.

The surfaces involved are smooth and white, their edges sharp. The incisors and left canine present a loss of substance absolutely typical. Gautrelet's analysis of the saliva in this case gave 0.200 gramme sulphocyanide of ammonium per litre. The urinary analysis showed increased acidity; total acidity expressed in phosphoric acid, 5.08 grammes per litre, or two hundred and ninety-five per cent. Leucomaines abundant; traces of sulphocyanides and mucin; chlorides and urea abundant; phosphates diminished.

Fig. 2.

Case III., Fig. 3: Mrs. A., aged fifty years; superior incisors eroded and bevelled from top to bottom; pulp-cavity of lateral incisors opened; canines eroded in furrows; hyperæsthesia intense. Treated with antimony chloride. Salivary analysis not made, but tested in my laboratory, a notable increase of sulphocyanides was observed.

Case IV., Fig. 4: Baron J., aged forty-two years; left upper canine shows erosion with sharp edges. The first right premolar has an abraded surface and also a transverse groove near the gum, mechanically produced by the tooth-brush. The two erosions are superposed and painless. The analysis of the saliva, made by Gautrelet, is as follows:

| | Grammes per litre. |
|---|---|
| Total acidity in phosphoric acid | 2.200 |
| Normal | 0.000 |
| Alkaline sulphocyanides | 0.072 |
| Normal | 0.015 |
| Alkaline chlorides | 4.530 |
| Alkaline sulphates | 0.460 |
| Alkaline earthy phosphates | 0.880 |
| Peptone | trace. |
| Albumin | 0.340 |
| Leucomaines | abundant. |

Microscopical examination revealed abundant epithelial cells, cell nuclei, leucocytes, and bacteria. Urinary analysis revealed a decided increase in the acidity. Expressed as phosphoric acid it

Fig. 3.

was 2.27 grammes per litre, or one hundred and thirty-two per cent.; chlorine, urea, and phosphates were diminished.

Case V., Fig. 5: Mrs. B., aged forty years; characteristic beginning chemical erosion; rheumatic diathesis. Left upper incisors and right lower incisors involved. The saliva contains traces of ammonium sulphocyanide. No urinary analysis. Case incomplete.

---

## Figures and Tables

**Figure f1:**
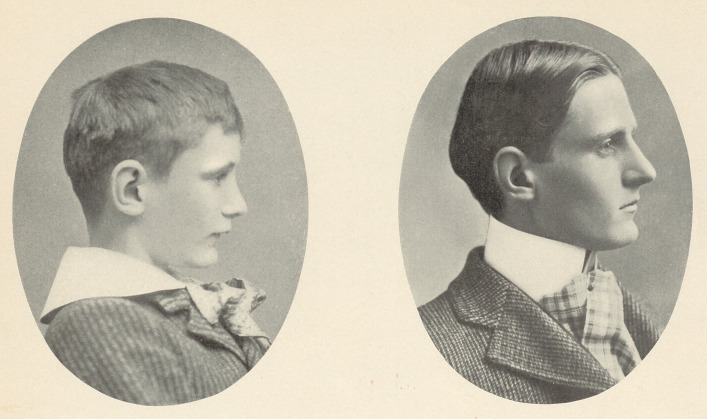


**Fig. 2. Fig. 1. Fig. 3. Fig. 4. Fig. 5. f2:**
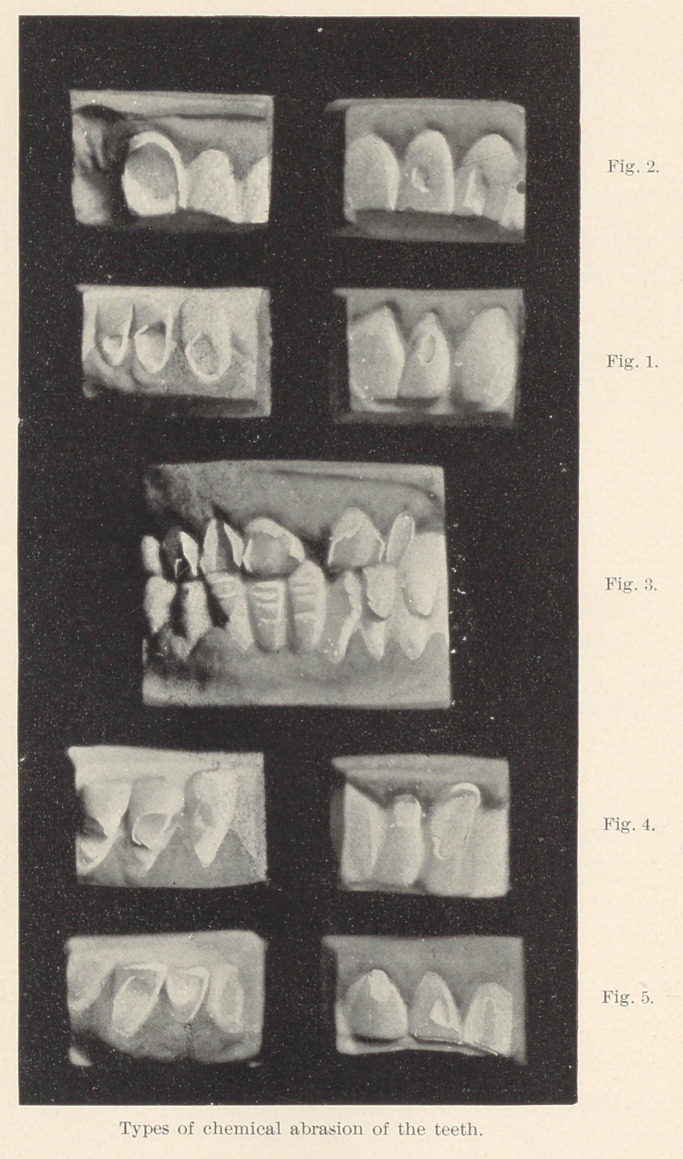


**Fig. 1. f3:**
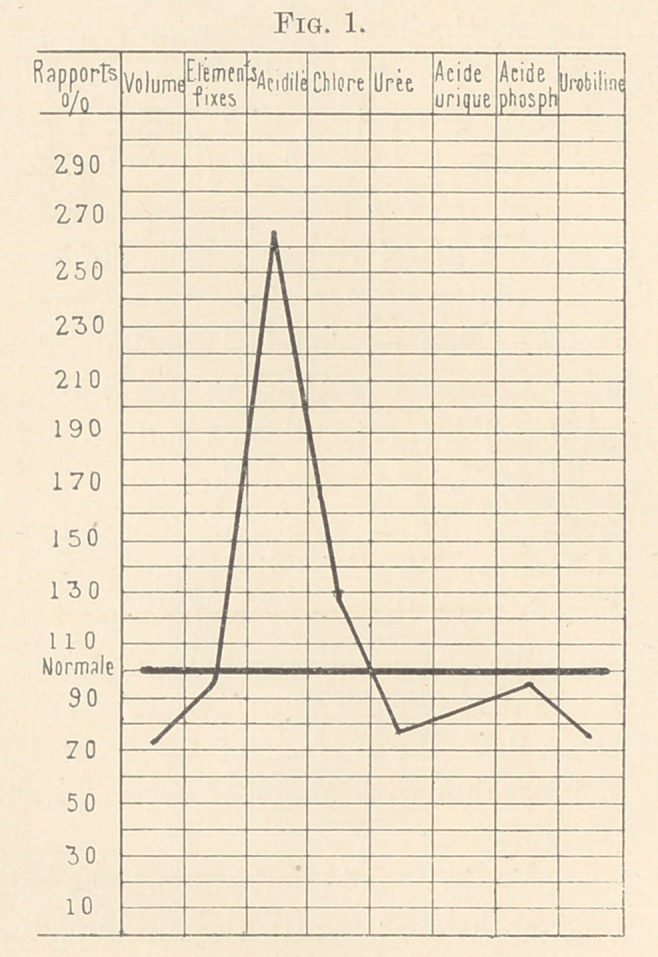


**Fig. 2. f4:**
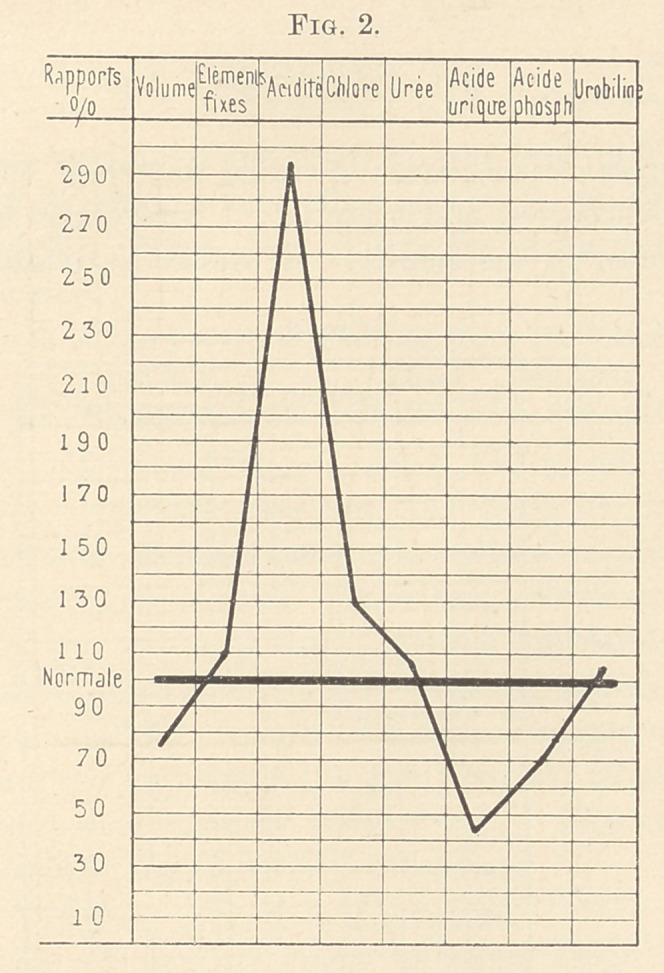


**Fig. 3. f5:**